# PTEN and ERG Biomarkers as Predictors of Biochemical Recurrence Risk in Patients Undergoing Radical Prostatectomy

**DOI:** 10.3390/diseases13080235

**Published:** 2025-07-24

**Authors:** Mihnea Bogdan Borz, Bogdan Fetica, Maximilian Cosma Gliga, Tamas-Csaba Sipos, Bogdan Adrian Buhas, Vlad Horia Schitcu

**Affiliations:** 1 Department of Anatomy, George Emil Palade University of Medicine, Pharmacy, Science, and Technology of Targu Mures, 540142 Târgu Mureș, Romania; borz.m.bogdan@gmail.com (M.B.B.);; 2 Department of Pathology, The Oncology Institute “Prof. Dr. Ion Chiricuţă”, 400015 Cluj-Napoca, Romania; 3 Department of Endocrinology, George Emil Palade University of Medicine, Pharmacy, Science, and Technology of Targu Mures, 540142 Târgu Mureş, Romania; 4 Urology Department, Faculty of Medicine and Pharmacy, University of Oradea, 410087 Oradea, Romania; 5 Urology Department, The Oncology Institute “Prof. Dr. Ion Chiricuţă”, 400015 Cluj-Napoca, Romania

**Keywords:** prostate cancer, radical prostatectomy, PTEN, ERG, immunohistochemistry, biochemical recurrence

## Abstract

**Background/Objectives**: Prostate cancer (PCa) remains a major global health issue, associated with significant mortality and morbidity. Despite advances in diagnosis and treatment, predicting biochemical recurrence (BCR) after radical prostatectomy remains challenging, highlighting the need for reliable biomarkers to guide prognosis and therapy. The study aimed to evaluate the prognostic significance of the PTEN and ERG biomarkers in predicting BCR and tumor progression in PCa patients who underwent radical prostatectomy. **Methods**: This study consisted of a cohort of 91 patients with localized PCa who underwent radical prostatectomy between 2016 and 2022. From this cohort, 77 patients were selected for final analysis. Tissue microarrays (TMAs) were constructed from paraffin blocks, and immunohistochemical (IHC) staining for PTEN and ERG was performed using specific antibodies on the Ventana BenchMark ULTRA system (Roche Diagnostics, Indianapolis, IN, USA). Stained sections were evaluated and correlated with clinical and pathological data. **Results:** PTEN expression showed a significant negative correlation with BCR (r = −0.301, *p* = 0.014), indicating that reduced PTEN expression is associated with increased recurrence risk. PTEN was not significantly linked to PSA levels, tumor stage, or lymph node involvement. ERG expression correlated positively with advanced pathological tumor stage (r = 0.315, *p* = 0.005) but was not associated with BCR or other clinical parameters. **Conclusions:** PTEN appears to be a valuable prognostic marker for recurrence in PCa, while ERG may indicate tumor progression. These findings support the potential integration of PTEN and ERG into clinical practice to enhance risk stratification and personalized treatment, warranting further validation in larger patient cohorts.

## 1. Introduction

According to the Global Cancer Observatory (GLOBOCAN) 2022 data, prostate cancer remains the second most frequently diagnosed cancer and the fifth leading cause of cancer death among men worldwide, accounting for over 1.41 million new cases and approximately 375,000 deaths annually [1,2]. Despite advances in diagnosis and treatment, PCa spans a wide clinical spectrum—from indolent to aggressive forms—and predicting biochemical recurrence (BCR) after radical prostatectomy remains a key challenge. Biomarkers are essential for risk stratification and guiding postoperative management [3,4].

PTEN is a tumor suppressor gene whose loss promotes tumor progression, metastasis, and treatment resistance. It has been consistently linked with BCR in prostate cancer patients undergoing radical prostatectomy. ERG, a transcription factor commonly overexpressed due to TMPRSS2-ERG fusion, is associated with early invasion and aggressive pathological features. While ERG’s individual prognostic role remains debated, its interplay with PTEN status may amplify risk, particularly in ERG+/PTEN− tumors. Together, they may serve as a complementary biomarker panel for recurrence prediction [5,6].

While several studies have explored PTEN or ERG individually as prognostic indicators [5,6], including earlier reports highlighting their expression heterogeneity in prostate biopsies and implications for sampling strategies [7], the novelty of this study lies in the combined immunohistochemical analysis of both markers in a well-defined, post-radical prostatectomy cohort with long-term follow-up. Unlike previous investigations, we assess the complementary predictive utility of PTEN and ERG in recurrence risk stratification, which may aid in postoperative decision-making. Prior studies often lacked integration of both markers in the same statistical model or focused on diagnostic rather than prognostic settings [5,6,8].

Prostate needle biopsy remains the gold standard for diagnosing prostate adenocarcinoma. However, pathologists often face challenges in interpreting biopsy specimens, especially when evaluating a small number of suspicious glands or minimal cellular atypia [9,10].

Immunohistochemistry (IHC) with markers such as high-molecular-weight cytokeratins, p63, epithelial cell adhesion molecule (EPCAM), transforming growth factor (TGF)-beta, and alpha-methylacyl-CoA racemase (AMACR) helps assess the integrity of the basal cell layer, the myoepithelial layer, and its infiltration by the tumor. Additionally, markers such as GalNac-T3 (N-acetylgalactosaminyltransferase 3), prostate-specific membrane antigen (PSMA), hepsin, and prostate cancer antigen 3 (PCA3) assist in differentiating PCa from benign prostatic hyperplasia. These biomarkers are crucial in diagnosing prostate carcinoma, evaluating invasiveness, and predicting responses to androgen therapy [9,10,11].

Despite extensive research on PCa biomarkers [12], there is an ongoing need to discover and implement novel biomarkers for patients who have undergone radical prostatectomy. These biomarkers could help differentiate aggressive forms of prostate cancer, guide personalized follow-up, and inform adjuvant treatment strategies, ultimately improving patient outcomes.

The biomarkers analyzed in this study are phosphatase and tensin homolog (PTEN) and ERG (ETS-related gene), which have shown consistent prognostic significance in localized prostate cancer [5,13,14,15,16,17,18,19]. PTEN is a tumor suppressor gene frequently lost in prostate cancer, and its loss has been associated with disease progression, metastasis, and poor prognosis. ERG, a transcription factor, is commonly overexpressed due to TMPRSS2-ERG gene fusion and has been linked to aggressive tumor behavior. Focusing on post-radical prostatectomy patients, this analysis seeks to refine recurrence risk stratification and enhance the effectiveness of subsequent therapeutic interventions. While previous studies have explored these biomarkers both individually and together, our study aims to evaluate their combined prognostic value specifically in predicting BCR and tumor progression.

## 2. Materials and Methods

### 2.1. The Study Population

Out of a total of 457 patients from the institute’s database who underwent surgery for clinically localized prostate cancer at our clinic between 2016 and 2022, 91 cases were selected based on the inclusion criteria for this study. The inclusion criteria were patients diagnosed with localized prostate cancer and a histopathological variant of prostate adenocarcinoma in the radical prostatectomy specimen, ISUP grade 1–4, negative surgical margins, an initial PSA up to 20 ng/mL, and a mandatory two-year follow-up. Of the selected cases, 11 prostatectomy paraffin blocks were not available, as they had been requested by patients for external analysis. For financial reasons, we initially planned to construct four microarray paraffin blocks, each containing 20 cases. However, 3 cases were lost during the construction process, leaving 77 cases for analysis with the immunohistochemical markers PTEN and ERG. BCR was defined as two consecutive postoperative PSA values greater than 0.2 ng/mL. This subgroup of patients reflected the biochemical recurrence (BCR) rates in the original database (31.1% vs. 27.3%).

### 2.2. Ethics Committee Approval and Data Collection

This study was conducted in accordance with the Declaration of Helsinki and was approved by the Ethics Committee at the “Prof. Dr. Ion Chiricuță” Oncology Institute, Cluj Napoca (approval number 388/4 September 2024).

### 2.3. Statistical Analysis

Pearson’s chi-square and Fisher’s tests were used to interpret categorical variations. Non-normally distributed continuous variables were compared using the Mann–Whitney test. The Kolmogorov–Smirnov and Shapiro–Wilk tests were employed to assess the normality of data distributions. The Kaplan–Meier method was used to analyze BCR. IBM SPSS v26 software was used to perform the statistical analysis. A *p*-value of less than 0.05 was established as statistically significant.

Pearson correlation analysis was employed to explore the linear association between continuous variables, such as biomarker expression scores and clinical/pathological factors. This allowed us to detect trends, such as decreasing PTEN expression with rising BCR risk or tumor grade, thereby providing early statistical insights into biomarker behavior.

### 2.4. Tissue Microarray Construction

The tissue microarray was constructed using a specialized TMA construction kit (Hisztopatológia Kft., Pecs, Hungary). The kit included a two-piece matrix (a base with 20 pins and a top with an insert for a block holder cassette), two lifting screws, and a “pen” perforation extractor. The matrix allowed for the creation of a paraffin recipient block containing 20 holes, each 2.0 mm in diameter, arranged in a 5 × 4 layout.

The protocol used for TMA construction in our study follows standard practices in research pathology laboratories. While core sampling techniques and matrix systems may vary by institution, the method employed here is commonly used in biomarker validation studies. Estimated material costs (excluding labor and equipment) for constructing each TMA block were approximately 100–150 EUR, depending on reagent brands and core density. These procedures are feasible in most academic pathology settings with appropriate histopathology infrastructure.

### 2.5. Preparation of Recipient TMA Paraffin Block

The preparation of the paraffin block was carried out in the special matrices using the protocol recommended by the manufacturer. The top matrix was aligned flat against the base using two guide pins, and a block holder cassette was placed in the matrix insert. The mold and cassette were then filled with liquid paraffin at a temperature of 56–60 °C and left to cool at room temperature for 20–30 min to ensure adequate hardening. The use of forced cooling methods, such as freezers or cold plates, was avoided to prevent potential block cracking. Once the paraffin had fully hardened, the matrix was lifted by slowly rotating the lifting screws clockwise. The resulting paraffin recipient block contained 20 holes, each 2.0 mm in diameter, arranged in five rows and four columns.

### 2.6. Obtaining Tissue Cores and Constructing the TMA Block

Tumor areas of interest were identified on previously stained tissue sections and corresponding donor blocks. To facilitate the core extraction, the donor blocks were re-embedded, providing additional paraffin above the cores to prevent tissue loss during sectioning. Tissue cores were extracted using the perforation extractor, which works with a spring-loaded retraction action. The extractor was pressed perpendicularly into the selected area of the donor block with a gentle twisting motion to a depth of approximately 4.0 mm. The tissue core was extracted at the tip of the perforation extractor.

The extracted tissue cores were then transferred to the recipient TMA block. The tip of the perforation extractor was aligned perpendicularly over the selected hole, and the piston was pressed firmly to insert the core without damaging the block. Proper technique ensured that the inserted cores were flush with the block surface. For accurate sampling, at least two cores from each representative area were extracted, ensuring correct identification of the tissue samples.

The filled TMA block was then placed in an oven at 56–58 °C for 10–15 min to recast the paraffin. Four new paraffin blocks were obtained, including 77 cases. These were subsequently sectioned into consecutive 2–4 µm sections, which were prepared for immunohistochemical analysis.

### 2.7. Immunohistochemical Stainings

Immunohistochemical staining was performed using the Ventana BenchMark ULTRA system (Roche Diagnostics, Indianapolis, IN, USA) in accordance with the manufacturer’s protocols. The primary antibodies employed in this study included the monoclonal antibody PTEN (clone 6H2.1), sourced from mice and produced by Diagnostic BioSystem, and the monoclonal antibody ERG (clone EP111), sourced from rabbits and produced by Dako Omnis. These antibodies are widely recognized markers in the diagnosis and study of prostate cancer.

The sections underwent a thorough deparaffinization and rehydration process, followed by antigen retrieval facilitated by the Ventana BenchMark ULTRA system. This automated system ensures consistency and accuracy during the staining process. Visualization was achieved using the DAB (3,3′-Diaminobenzidine) chromogen, with subsequent counterstaining using hematoxylin.

To evaluate the expression of PTEN and ERG, stained sections were analyzed under a light microscope. The assessment was based on a standardized scoring system, where staining intensity was graded on a scale from 0 to 3, and the percentage of tumor cells exhibiting positive staining was recorded. Cases were considered positive if they met a predefined threshold for both intensity and percentage of positivity. The results were systematically analyzed for correlation with clinical and pathological characteristics of prostate cancer, employing statistical methods such as regression analysis to support our findings.

**Figure 1:**
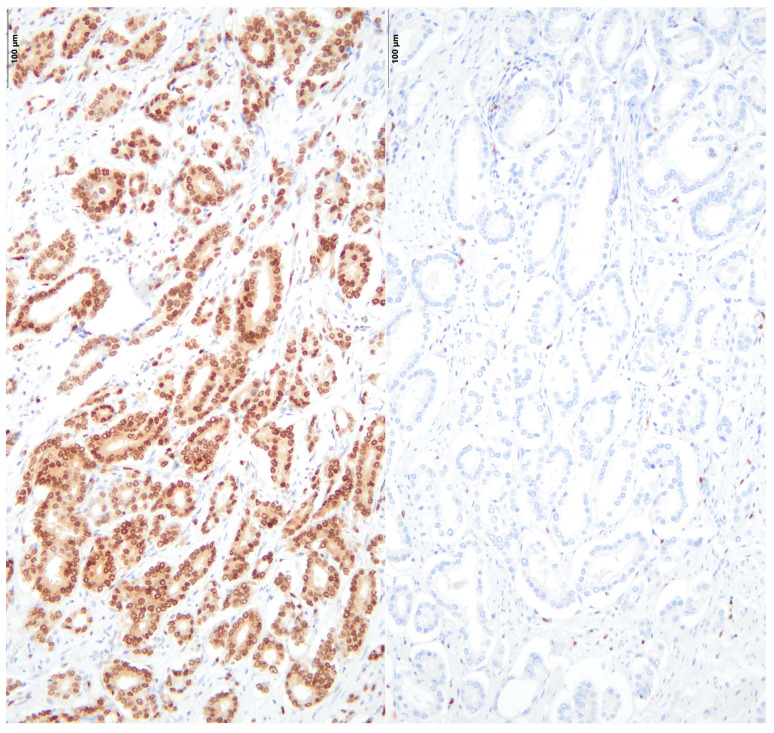
Positive (**left**) and negative (**right**) ERG protein expression in radical prostatectomy specimens, seen under light microscope.

**Figure 2:**
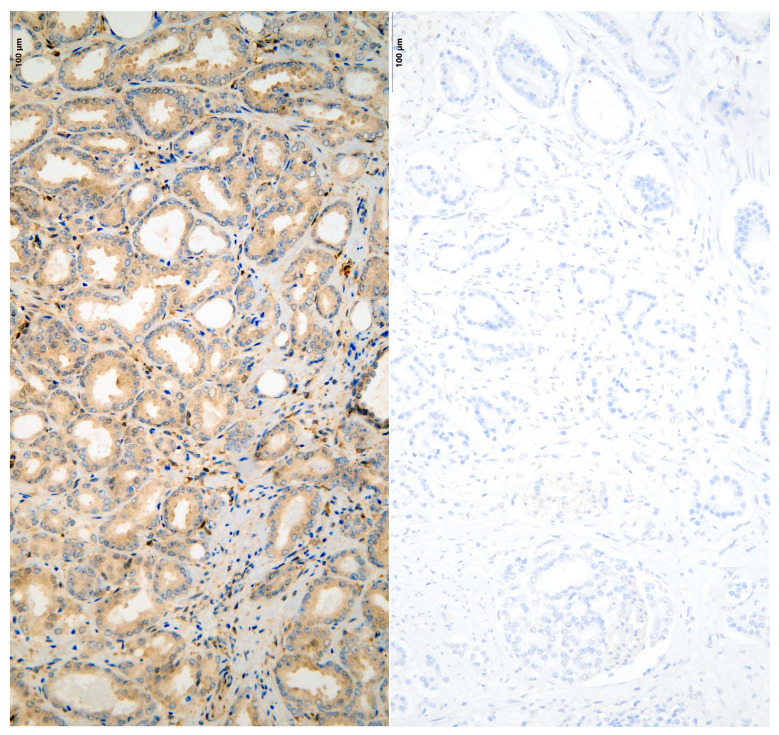
Positive (**left**) and negative (**right**) protein expression of cytoplasmically labeled PTEN in radical prostatectomy specimens, seen under light microscope.

Figure 1 and Figure 2 illustrate sections from the TMA blocks stained for ERG and PTEN. The images depict both positive expression (right) and negative expression (left). It is important to note that the staining intensity for PTEN in the “positive” cases, while demonstrating some level of expression, may appear weak.

The IHC antibodies (PTEN clone 6H2.1 and ERG clone EP111) used in this study are clinically validated and widely used in diagnostic pathology settings. Despite subjective variation, the scoring was performed independently by two experienced pathologists blinded to clinical data, ensuring consistency.

## 3. Results

To facilitate meaningful statistical analysis, the cohort of 77 patients was stratified into subgroups based on their immunohistochemical staining results for PTEN and ERG. Each biomarker was assessed independently, and patients were categorized as either positive or negative for expression based on the presence or absence of specific staining patterns in tumor cells. This binary classification enabled the comparison of clinical and pathological characteristics between expression groups, allowing evaluation of potential correlations between biomarker status and outcomes such as biochemical recurrence and tumor progression.

To investigate potential relationships between PTEN gene expression and various clinical and pathological characteristics, we conducted a correlation analysis using Pearson’s correlation coefficient. The variables analyzed included iPSA levels, pathological tumor staging (pT), pathological nodal involvement (pN), ISUP grading, and BCR (Table 1).

The analysis revealed that PTEN expression was weakly and negatively correlated with iPSA (r = −0.074, *p* = 0.55) and pT staging (r = −0.063, *p* = 0.61). Similarly, the correlation between PTEN expression and pN staging was weak and negative (r = −0.097, *p* = 0.43), indicating no significant association with lymph node involvement in this cohort.

For ISUP grading, a weak negative correlation with PTEN expression was observed (r = −0.239, *p* = 0.053). Although this result approached statistical significance, it did not meet the conventional *p* < 0.05 threshold, suggesting a potential trend where lower PTEN expression might be associated with higher ISUP grades.

Notably, a moderate negative correlation was identified between PTEN expression and BCR (r = −0.301, *p* = 0.01), which was statistically significant. This finding suggests that reduced PTEN expression is associated with a higher likelihood of biochemical recurrence, supporting its potential role as a prognostic marker.

To further explore the relationship between PTEN expression and various clinical and pathological variables, a logistic regression analysis was performed. PTEN expression was dichotomized based on its median value, and the model included iPSA levels, pathological tumor staging (pT), pathological nodal staging (pN), ISUP grade, and BCR as independent variables.

The results indicated a statistically significant association between PTEN expression and risk of BCR. Specifically, the odds ratio for BCR was 0.280 (95% CI: 0.07, 1.10; *p* = 0.01), suggesting that patients with biochemical recurrence were approximately 72% less likely to exhibit high PTEN expression compared to those without recurrence.

**Figure 3:**
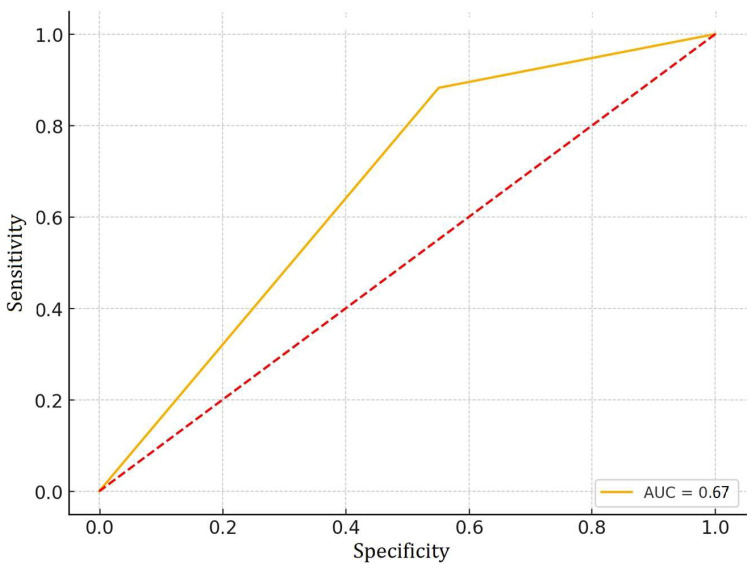
ROC curve for prediction of biochemical recurrence in relation to PTEN.

To evaluate the ability of PTEN expression to predict BCR, we generated a Receiver Operating Characteristic (ROC) curve (Figure 3).

The area under the curve (AUC) was calculated to quantify the model’s performance. The resulting AUC value was 0.67, indicating modest predictive accuracy. This result suggests that negative PTEN expression may be associated with an increased risk of BCR.

A Pearson correlation analysis was performed to explore the relationship between ERG expression and various clinical and pathological variables, including iPSA levels, pT, pN, ISUP grade, and BCR (Table 2).

The analysis revealed a statistically significant moderate positive correlation between ERG expression and pT stage (r = 0.315, *p* = 0.03), suggesting an association between higher ERG expression and an advanced pathological T stage. This finding may indicate a potential role for ERG in tumor progression.

No significant correlations were observed between ERG expression and the other variables. Specifically, the correlations between ERG and iPSA (r = 0.016, *p* = 0.97), pN (r = 0.122, *p* = 0.33), ISUP grade (r = 0.157, *p* = 0.29), and BCR (r = 0.145, *p* = 0.27) were weak and not statistically significant.

A logistic regression analysis was conducted to assess the relationship between multiple predictor variables (iPSA, pT, pN, ISUP grade, BCR) and the likelihood of ERG positivity. The model was statistically significant (*p* < 0.05), indicating that this group of variables can reliably differentiate between ERG-positive and ERG-negative outcomes.

The analysis indicated that the pT variable was significantly associated with ERG positivity. Specifically, the odds of ERG positivity increased by approximately 5.18 times for each unit increase in pT (OR = 5.18, 95% CI [1.12, 23.94], *p* < 0.05). These findings suggest that higher pT levels are strong predictors of ERG positivity.

In contrast, the other variables, including iPSA, pN, ISUP grade, and BCR, did not show statistically significant associations with ERG positivity.

**Figure 4:**
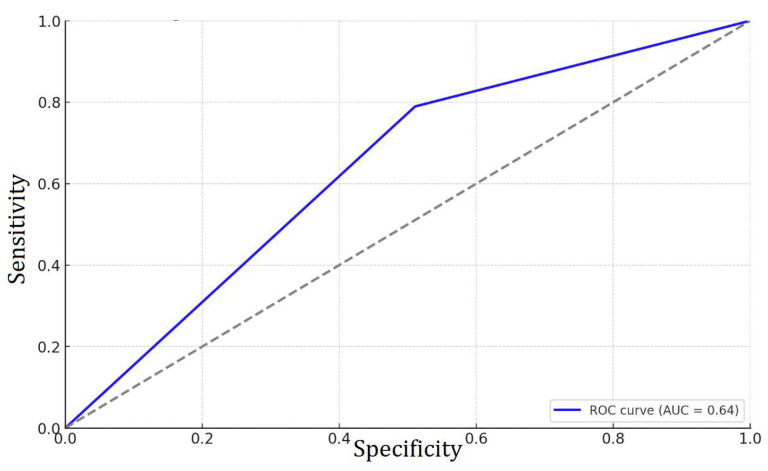
ROC curve for tumor stage prediction in relation to ERG.

The ROC curve (Figure 4) was plotted to predict binary pT using ERG, and the area under the curve (AUC) is approximately 0.64. This value indicates that ERG has a modest ability to predict whether pT is above or below the median value.

## 4. Discussion

This study investigated PTEN and ERG expression in PCa and their associations with clinical and pathological features. While these biomarkers have been extensively studied, including prior analyses confirming their prognostic value in prostate cancer [20], our findings reinforce their significance in a Romanian cohort undergoing radical prostatectomy—an underrepresented population in the current literature. By focusing on biochemical recurrence (BCR) as a clinical endpoint, our data highlight the translational potential of PTEN and ERG for postoperative risk stratification.

A key limitation of this study is the semi-quantitative nature of immunohistochemistry, which—though widely used—may lack the precision of molecular assays. We addressed potential interobserver variability by applying standardized cutoffs and ensuring independent scoring by two pathologists.

PTEN loss is a well-established event in PCa and has been associated with poor prognosis, including higher tumor grade and increased BCR risk [6]. Our results confirmed a significant negative association between PTEN expression and BCR (r = –0.301, *p* = 0.01), consistent with previous findings in radical prostatectomy cohorts [8,18,21,22,23,24,25,26,27,28]. Although the association with ISUP grade was only borderline significant (r = –0.239, *p* = 0.053), the trend suggests a possible link with tumor aggressiveness that warrants further investigation in larger cohorts.

We found no significant associations between PTEN expression and iPSA, tumor stage (pT), or lymph node involvement (pN), consistent with some but not all previous studies [29]. These discrepancies may reflect differences in methodology or sample composition.

Logistic regression confirmed a significant association between reduced PTEN expression and BCR risk (OR = 0.280, *p* = 0.01). ROC analysis showed modest discriminative ability (AUC = 0.67), reinforcing PTEN’s potential as a prognostic marker, though its standalone predictive value remains limited. The observed pattern aligns with previous findings, suggesting a potential role for PTEN in recurrence prediction [6,27,28,30].

Due to the limited number of events, we did not perform multivariate modeling to avoid overfitting. Future larger-scale studies should incorporate multivariate analysis to adjust for confounders such as ISUP grade, surgical margin status, and lymph node involvement.

ERG rearrangements and overexpression have been frequently reported in PCa, with some studies linking ERG positivity to more aggressive disease and poorer outcomes [3,8,19,24,29]. In our cohort, ERG expression showed a significant association with advanced pathological stage (r = 0.315, *p* = 0.03), consistent with its role in tumor progression [5,26].

Our findings are broadly consistent with previous studies highlighting the prognostic relevance of PTEN and ERG, though variations in study cohorts and methodologies may account for differences in observed associations. For example, Schaefer et al. reported distinct ERG rearrangement patterns, with higher prevalence in younger patients and those with lower PSA levels, suggesting heterogeneity in ERG-related tumor biology [31]. Similarly, Alshalalfa et al. emphasized the genomic complexity of metastatic PCa, including alterations in ERG and PTEN pathways, which may influence recurrence risk differently in localized versus advanced settings [32]. Shore et al. also underlined the role of biomarker-based risk stratification in tailoring post-treatment monitoring and adjuvant therapy [33]. These findings underscore the need to interpret PTEN and ERG prognostic roles in the context of disease stage and clinical variables.

In our cohort, ERG expression was not significantly associated with biochemical recurrence but correlated with advanced pathological stage. This partially diverges from some previous observations, possibly due to differences in population, sample size, or scoring thresholds [34,35]. Future biomarker panels may benefit from integrating ERG/PTEN with additional molecular features such as chromogranin levels or DNA repair gene alterations.

In contrast to some earlier studies, we did not observe significant associations between ERG expression and iPSA, pN, ISUP grade, or BCR. This inconsistency likely reflects methodological and population differences across studies [5,22,25,30,36]. Our findings suggest that ERG may be more closely associated with tumor stage than with recurrence risk.

Logistic regression further supported the association between ERG positivity and advanced tumor stage (OR = 5.18, *p* < 0.05). ROC analysis yielded an AUC of 0.64, indicating modest predictive performance. These modest AUC values for both PTEN (0.67) and ERG (0.64) highlight the limited clinical utility of these biomarkers when used in isolation.

Taken together, our findings suggest that while ERG expression reflects tumor advancement, its utility for predicting BCR is limited. Notably, we did not assess the prognostic value of combined PTEN/ERG alteration status (e.g., PTEN−/ERG+), which has been proposed to exert a synergistic effect on recurrence risk [6,8]. Future studies should explore this interaction more deeply to clarify the potential of integrated biomarker panels.

A final limitation of our study is the relatively small sample size (n = 77), which may have limited the power to detect subtle associations, especially for ERG expression. Larger multicenter cohorts are needed to validate these results and explore subgroup interactions more robustly.

Although our data support the clinical relevance of PTEN and ERG, more comprehensive molecular characterization is necessary. Incorporating transcriptomic or proteomic analyses—particularly those targeting downstream effectors such as the PI3K/AKT pathway—could provide deeper insights into the biological mechanisms driving recurrence. These approaches may ultimately refine biomarker-guided patient stratification and enable more personalized therapeutic strategies.

## 5. Conclusions

Our findings indicate that PTEN and ERG biomarkers may have potential utility in assessing risk and guiding personalized treatment strategies in prostate cancer patients following radical prostatectomy. Specifically, reduced PTEN expression was associated with an increased risk of BCR, supporting its role as a potential prognostic marker. ERG expression, on the other hand, was correlated with higher pathological tumor stage, suggesting its relevance in evaluating tumor progression. Despite promising results, these conclusions must be interpreted with caution due to the study’s limitations.

To support the clinical implementation of these biomarkers, future studies should focus on large-scale, multi-institutional cohorts to validate these associations across diverse populations. Additionally, systematic reviews and meta-analyses are essential to synthesize existing data and determine the strength and consistency of these biomarkers’ predictive value.

The path to clinical translation will also require standardized evaluation protocols and integration into multi-parametric risk models. This approach could improve their clinical utility by combining molecular, pathological, and clinical data for a more comprehensive assessment of patient risk.

Ultimately, while our study contributes to the growing body of evidence on the prognostic relevance of PTEN and ERG, further high-quality research is necessary before these biomarkers can be included in clinical guidelines or used in routine clinical decision-making. Continued investigation is needed to ensure that their application contributes meaningfully to improved outcomes and more personalized care for patients with prostate cancer.

## Figures and Tables

**Table 1:** Univariate analysis of factors influenced by the PTEN expression.

Variables				
			95% C.I. for OR	
	*p*	Odds Ratio	Inferior	Superior
iPSA	0.55	1.03	0.87	1.22
pT	0.61	1.04	0.27	3.99
pN	0.43	1.51	0.10	2.99
ISUP grade	0.053	0.63	0.32	1.24
BCR	0.01	0.28	0.07	1.10

iPSA = initial PSA, pT = tumor stage, pN = lymph nodes stage, ISUP grade = The International Society of Urological Pathology grade, BCR = biochemical recurrence.

**Table 2:** Univariate analysis of factors influenced by the presence of ERG.

Variables				
			95% C.I. for OR	
	*p*	Odds Ratio	Inferior	Superior
iPSA	0.97	0.88	0.74	1.05
pT	0.03	5.17	1.11	23.94
pN	0.33	1.27	0.09	17.13
ISUP grade	0.29	1.24	0.68	2.26
BCR	0.27	2.57	0.60	10.90

iPSA = initial PSA, pT = tumor stage, pN = lymph nodes stage, ISUP grade = The International Society of Urological Pathology, BCR = biochemical recurrence.

## Data Availability

Further data and the datasets supporting this study are available from the corresponding author upon justified demand.

## Abbreviations

The following abbreviations are used in this manuscript:

AMACR	alpha-methylacyl-CoA racemase
AUC	Area Under the Curve
BCR	Biochemical Recurrence
CI	Confidence Interval
DAB	3,3′-Diaminobenzidine
EPCAM	Epithelial Cell Adhesion Molecule
ERG	ETS-related gene
GalNac-T3	N-acetylgalactosaminyltransferase 3
IHC	Immunohistochemistry
ISUP grade	International Society of Urological Pathology grade
iPSA	Initial Prostate-Specific Antigen
OR	Odds Ratio
PCa	Prostate Cancer
PCA3	Prostate Cancer Antigen 3
PSMA	Prostate-Specific Membrane Antigen
PTEN	Phosphatase and Tensin Homolog
ROC	Receiver Operating Characteristic
SPSS	Statistical Package for the Social Sciences
TGF-beta	Transforming Growth Factor-beta
TMA	Tissue Microarray
